# Cinnamon Aqueous Extract Attenuates Diclofenac Sodium and Oxytetracycline Mediated Hepato-Renal Toxicity and Modulates Oxidative Stress, Cell Apoptosis, and Inflammation in Male Albino Rats

**DOI:** 10.3390/vetsci8010009

**Published:** 2021-01-06

**Authors:** Gehad E. Elshopakey, Sara T. Elazab

**Affiliations:** 1 Department of Clinical Pathology, Faculty of Veterinary Medicine, Mansoura University, Mansoura 35516, Egypt; 2 Department of Pharmacology, Faculty of Veterinary Medicine, Mansoura University, Mansoura 35516, Egypt; Sara.taha@ymail.com or

**Keywords:** cinnamon, diclofenic sodium, oxytetracycline, hepato-renal toxicity, oxidative stress, immunohistochemistry

## Abstract

Among commonly consumed anti-inflammatory and antimicrobial drugs are diclofenac sodium (DFS) and oxytetracycline (OTC), especially in developing countries because they are highly effective and cheap. However, the concomitant administration of anti-inflammatory drugs with antibiotics may exaggerate massive toxic effects on many organs. Cinnamon (*Cinnamomum zeylanicum*, Cin) is considered one of the most broadly utilized plants with various antioxidant and anti-inflammatory actions. This study aimed to evaluate the possible protective effects of cinnamon aqueous extract (Cin) against DFS and OTC hepato-renal toxicity. Eight groups (8/group) of adult male albino rats were treated orally for 15 days with physiological saline (control), Cin aqueous extract (300 mg/kg b.w.), OTC (200 mg/kg b.w.), single dose of DFS at the 14th day (100 mg/kg b.w.), DFS + OTC, Cin + DFS, Cin + OTC, and Cin + DFS + OTC. The administration of DFS and/or OTC significantly increased (*p* < 0.05) the serum levels of alanine aminotransferase, aspartate aminotransferase, alkaline phosphatase, urea, creatinine, and uric acid. Serum levels of pro-inflammatory cytokines, as well as hepatic and renal malondialdehyde and nitric oxide metabolites, were also raised following DFS and OTC administration. Meanwhile, the activities of reduced glutathione, superoxide dismutase, and catalase in liver and kidney were significantly suppressed in DFS, OTC, and DFS + OTC treated rats. Moreover, hepatic and renal tissue sections from these rats exhibited overexpression of caspase-3 and cyclooxygenase-II on immunohistochemical investigation. The administration of Cin aqueous extract ameliorated the aforementioned deteriorations caused by DFS, OTC, and their combination. Conclusively, Cin is a promising protective plant extract capable of attenuating the oxidative damage, apoptosis, and inflammation induced by DFS and OTC either alone or combined, on hepatic and renal tissues.

## 1. Introduction

Diclofenic sodium (DFS), a nonsteroidal anti-inflammatory (NSAID), analgesic and antipyretic drug, is widely used globally to relieve pain and fever [1,2]. It is extensively consumed for the management of many chronic conditions such as rheumatoid arthritis, osteoarthritis, ankylosing spondylitis [3]. DFS elicits its action by hindering the formation of prostaglandin through suppressing cyclooxygenase-1 (Cox-I) and cyclooxygenase-2 (Cox-II) enzymes with the same potency [4].The presence of DFS as an over-the counter drug may lead to its abuse resulting in threatening deleterious effects on the liver, kidney, and gastrointestinal tract [5]. The misuse of DFS has been contributed to gastrointestinal problems [6], hepatotoxicity [7,8,9], nephrotoxicity [10], and neurotoxicity [11]. Oxidative stress and mitochondrial damage are the main elements collaborating in DFS toxicity [12,13]. Non-steroidal anti-inflammatory drugs (NSAIDs) are commonly consumed in combination with antibacterial medications. However, the concomitant administration of NSAIDS with antibiotics may exaggerate the toxic effects of both drugs. Therefore, the effect of combining DFS and OTC on biological systems needs to be evaluated.

Oxytetracycline (OTC), a member of tetracycline antibiotics, possesses high activity against a wide variety of micro-organisms, including Gram-positive and Gram-negative bacteria, in addition to chlamydia, rickettsia, mycoplasmas, and protozoan parasites [14]. It is commonly prescribed for the management of respiratory and skin diseases in human and livestock, particularly in developing countries for its antimicrobial efficacy and low cost [15]. The irrational utilization of OTC in excessive doses without medical guidance has harmful effects on the liver and kidney [15]. Prior report has shown that toxic doses of OTC caused vigorous microvesicular steatosis and even hepatic damage [16]. It has been recorded that OTC exhibits its hepato-renal toxicity through induction of membrane lipid peroxidation and reduction of tissue antioxidants biomarkers [17].

Several herbal medications are being evaluated for their mitigative effects and antioxidant activity [18,19]. Recently, great attention has been paid to the use of herbal remedies to treat many diseases [20]. Cinnamon (*Cinnamomum zeylanicum*, Cin) is considered one of the most broadly utilized plants in herbal treatment with various bioactive actions. It acts as a source of natural antioxidant for enhancing human health [21]. Cinnamon is characterized by its high content of polyphenolic compounds which serve as free radical scavengers [22]. Several pharmacological effects were recorded for cinnamon as anti-inflammatory, anti-microbial, anti-oxidant, and anti-diabetic effects [23,24,25]. It has been reported that total cinnamon extract may guard against cadmium, glutamate, bisphenol, gentamicin-induced oxidative injury [24,25,26,27].

This study was designed to assess whether DFS exaggerates the hepatorenal toxicity of OTC and whether cinnamon can guard hepatic and renal tissues against the hepatorenal toxicity caused by DFS and OTC.

## 2. Material and Methods

### 2.1. Plant Extract Preparation

The cinnamon extract was prepared according to the method of Abdeen et al. [24] with some modifications. Dry cinnamon (*Cinnamomum zeylanicum*) bark was purchased and identified from Faculty of Agriculture, Mansoura University. Briefly, 10 g of cinnamon bark was cleaned, grounded with a mechanical grinder to form a fine powder. The powder was soaked in distilled water (100 mL). The mixture was boiled at 100 °C for 2 h. Then, it was filtered and dried overnight by heating in an oven at 80 °C. The resultant dry extract was weighed and kept for further analyses and administration. The yield percentage of crude cinnamon bark aqueous extract was about 20% (*w*/*w*).

### 2.2. Phytochemical Analysis

Total phenolic content was performed according to a previous study by Wolfe et al. [28], using the Folin–Ciocateu’s reagent. Total flavonoid content was predestined following the protocol of Okada et al. [29] by using the aluminum chloride colorimetric method. The vanillin method was used to estimate total tannin content according to a previous report by Sadasivam and Manickam [30]. Additionally, the antioxidant activity was evaluated using the free radical scavenging method (DPPH) as reported by Thaipong et al. [31], then the maximum inhibitory concentration (IC_50_) was calculated using the exponential curve.

### 2.3. Chemicals

Oxytetracycline was purchased from Cid Co., El Haram, Giza, Egypt. Diclofenic sodium was procured from Novartis Pharma company (El Amireya, Cairo, Egypt).

### 2.4. Experimental Animals and Design

Sixty-four adult male albino rats weighing between 130–160 g were obtained from the laboratory animal unit of Zagazig University. During 14 days of acclimatization, the animals were fed normal control diet and water ad libitum. The protocol of this experiment was accepted by the Animal Ethical Committee of the Faculty of Veterinary Medicine, Mansoura University, Egypt (approval no. R/2). The animals were randomly divided into the following eight experimental groups (n = 8); the control group; received physiological saline daily. Cin groups; treated daily with Cin aqueous extract at a dose of 300 mg/kg b.w. for 15 days [32]. DFS group; received a single dose of DFS at 14th day (100 mg/kg b.w.) [33]. OTC group; rats received 200 mg/kg b.w./day of OTC for 15 days [34]. DFS + OTC group; received OTC for 15 days and a single oral dose of DFS at 14th day with the same previous doses. Cin + DFS group; rats received cinnamon extract for 15 days and treated with diclofenac sodium at the 14th day. Cin + OTC group; treated with cinnamon extract and 30 min later the rats received OTC for 15 days. Cin + DFS + OTC group; treated with a combination of Cin extract, DFS and OTC, at the same previous manner and doses. All rats were treated orally according to their group using a stomach tube.

### 2.5. Samples Collection

**Figure 1:**
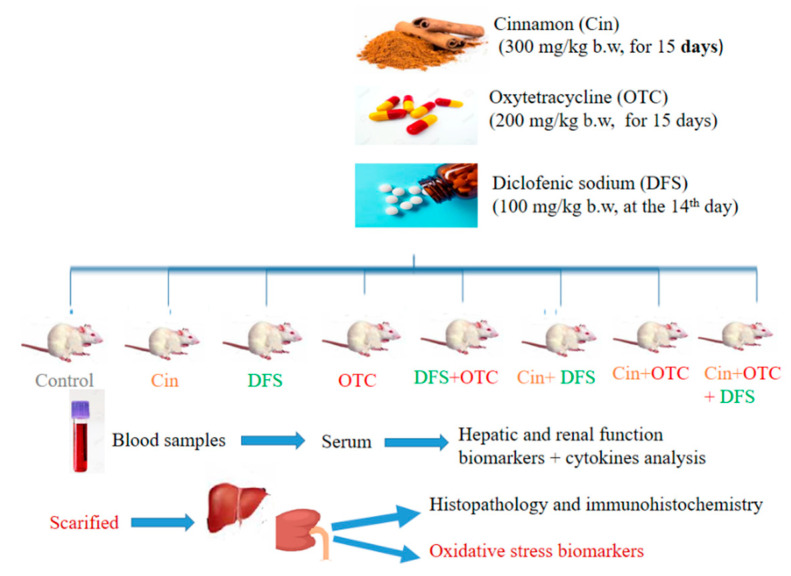
Study diagram and treatment protocol (Cin, Cinnamon; DFS, Diclofenic sodium; OTC, Oxytetracycline).

At the end of experiment, the blood samples were collected in a plane test tube from the retro-orbital venous sinus of each animal. Then, they were left 10 min to clot and centrifuged at 3000 rpm (4 °C) for serum separation. The separated serum was stored at –80 °C for further determination of biochemical and inflammatory biomarkers. Later, rats were anesthetized and sacrificed by cervical dislocation to collect liver and kidney tissues from each rat. One gram of liver and kidney tissues were previously washed three times using cold NaCl solution (0.9%) and homogenized in cold phosphate-buffered saline (PBS) (9 mL; PH 7.5) [35]. The liver and kidney homogenates were cold centrifuged for about 15 min at 3000 rpm and the supernatants were carefully collected in a clean tube to be used in the evaluation of antioxidants and oxidative stress parameters. A small portion from each organ was preserved in 10% neutral-buffered formalin for histopathological and immunohistochemical assessment. Study diagram and treatment protocol are shown in Figure 1.

### 2.6. Biochemical Analysis

#### 2.6.1. Serum Liver and Kidney Markers

The activity of serum alanine and aspartate aminotransferases (ALT, cat. no. AL146 and AST, cat. no. AS101) (Randox, Crumlin, UK), alkaline phosphatase (ALP, cat. no. A504-150) (Teco diagnostics, Anaheim, CA, USA), total protein (cat. no. SB-0250-500) and albumin (cat. no. SB- 028-500) levels (Stanbio laboratory, Boerne, TX, USA), urea (cat. No. URE118200, BioMed, Cairo, Egypt), creatinine (cat. no. 10051, Human, Wiesbaden, Germany) and uric acid (cat. no. MD41001, Spinreact, Girona, Spain) were estimated spectrophotometrically (5010, Photometer, BM Co., Eiterfeld, Germany) according to manufacturer’s protocol in the enclosed pamphlets.

#### 2.6.2. Oxidative Stress and Antioxidant Markers

The homogenized hepatic and renal tissue samples were utilized to estimate the oxidative stress and antioxidant markers. Commercial test kits (Biodiagnostics Co., Cairo, Egypt) for malondialdehyde (MDA, cat. no. MD 25 29), glutathione (GSH, cat. no. GR 25 11), catalase (cat. no. CA 25 17) and superoxide dismutase (SOD, cat. no. SD 25 21) were used for this analysis.

The hepatic and renal nitric oxide (NO) level was detected using ready kits (Abcam, Co., Cambridge, MA, USA; ab65328) according to the manufacturer’s protocols. Briefly, the nitrate is catalyzed with nitrate reductase into nitrite. Later, total nitrite is converted into a deep purple azo compound (azo chromophore) with Griess Reagents. The absorbance of the purple azo compound is measured at 540 nm, where the absorbance of the azo compound is directly proportional to NO production. The detection limit of the assay is approximately 1 nmol nitrite/well, or 10 µM.

#### 2.6.3. Serum Immunoglobulin, *C-Reactive* Proteins (CRP) and Cytokines

Immunoglobulin M (IgM, REF; 035071190), Immunoglobulin G (IgG, REF; 03507432) and CRP (REF; 04956842) were measured using ELISA ready-made kits obtained from cobas company, USA. Serum tumor necrosis factor alpha (TNF-α, cat. no. STA00D), interleukins (IL-6, cat. no. S6050), IL-10 (cat. no. S1000B) and IL-12 (cat. no. S1200) levels were estimated using ELISA ready-made commercial kits (Quantikine Co., Minneapolis, MN, USA).

### 2.7. Histopathological Examination

Hepatic and renal tissues were kept in 10% formalin. Then, standard histological technique was applied including dipping in serial ascending dilution of ethanol. After that, the tissue specimens were embedded in paraffin. The obtained paraffin blocks were sectioned at 4 µm thickness and then the sections were stained with hematoxylin and eosin (H&E), as declared by Bancroft and Layton [36].

### 2.8. Immunohistochemistry

Caspase-3 and Cox-II immunohistochemical staining of hepatic and renal sections was carried out as illustrated by (Noreldin et al. 2016). In summary, the hepatic and renal sections were deparaffinized and rehydrated employing a serial of graded alcohol washes. The antigen retrieval using 10 mM citrate buffer (pH 6.0) for 10–20 min was performed for the sections prepared for anti-Cox-II, then the sections were preserved at room temperature for 20 min and rinsed with distilled water. The sections for caspase-3 did not expose to any antigen retrieval method. Inactivation of endogenous peroxidase was performed by 3% H_2_O_2_ in methanol 100% at 4 °C for 30 min followed by washing in PBS. The slides were blocked in10% normal blocking serum for 1 h at 25 °C. Then, the slides were incubated with anti-caspase-3 the primary antibody (polyclonal rabbit anti-cleaved caspase-3 at dilution 1:100, BioCare Medical, Cat: CP229C, Concord, California CA, USA) and anti-Cox-II (monoclonal rabbit anti-Cox-II at dilution 1:100; ThermoFisher Scientific, Cat: RM-9121-S0, Fremont, CA, USA). Thereafter, the slides were exposed to biotinylated goat anti-rabbit IgG antiserum (Histofine kit, Nichirei Corporation, Tokyo, Japan) for 60 min and they were washed with PBS. Finally, the streptavidin-peroxidase conjugate (Histofine kit, Nichirei Corporation) was added to the slides for 30 min. The reaction was visualized by treatment with 3, 3′-diaminobenzidine tetrahydrochloride (DAB)-H_2_O_2_ solution (pH 7.0) for 3 min. The slides were washed in distilled water and counterstained with hematoxylin.

### 2.9. Statistical Analysis

Data were expressed as mean ± standard error of the mean (SEM). The differences among biochemical, antioxidant and oxidative stress parameters; and inflammatory biomarkers were applied on the SPSS software program (version 20, USA) using a one-way analysis of variance (ANOVA) followed by Duncan multiple comparison tests. At *p* < 0.05, the differences were considered to be statistically significant.

## 3. Results

### 3.1. Phytochemical Components of Cinnamon Aqueous Extract

The total phenolic compounds showed the highest levels of the active constituents present in Cin aqueous extract, followed by flavonoids and tannin contents, respectively, as demonstrated in Table 1. The content of total phenolics in the extract was 10.45 mg gallic acid equivalent/mL. In addition, the flavonoid content was 4.76 mg quercetin equivalent/mL, while tannins contents were about 2.51 mg catechin equivalent/mL. The IC_50_ of the extract was estimated with respect to DPPH assay and constituted 43.58 mg/mL.

### 3.2. Serum Hepatic and Renal Injury Biomarkers

The serum ALT, AST, and ALP activities were significantly elevated in DFS + OTC, OTC and DFS groups unlike the control and Cin groups (*p* < 0.05). In addition, all of the previous parameters were dramatically reduced upon the treatment with Cin in the Cin + DFS + OTC, Cin + OTC and Cin + DFS groups compared to the DFS + OTC, OTC, and DFS groups, respectively, but still higher than that of the control and Cin groups (*p* < 0.05) (Table 2). Significantly reduced levels of total protein, albumin, and globulin were recorded in DFS-OTC and DFS groups comparing to the control one and Cin treated rats (*p* < 0.05). Meanwhile, total protein level was only significantly affected in OTC groups compared to the control one (*p* < 0.05). Cin treatment could ameliorate the toxic effect of DFS + OTC and DFS and caused a significant increase in total protein, albumin, and globulin levels of Cin + DFS + OTC and Cin + DFS groups when compared to DFS + OTC and DFS groups (*p* < 0.05). Only total protein was significantly increased in OTC and Cin + OTC groups, while albumin and globulin remain unaffected when compared to the control (*p* < 0.05) (Table 2).

The serum levels of urea, creatinine, and uric acid were significantly higher in DFS + OTC, OTC, and DFS groups than in the control and Cin groups (*p* < 0.05). The Cin + DFS + OTC, Cin + OTC and Cin + DFS treated groups exhibited the ability of Cin to modify renal toxicity by decreasing urea, creatinine, and uric acid serum levels in these groups compared to the DFS + OTC, OTC, and DFS groups (*p* < 0.05) (Table 3).

### 3.3. Hepatic and Renal Oxidative Stress and Antioxidant Markers

Hepatic and renal MDA and NO levels were significantly elevated in DFS + OTC, OTC and DFS treated rats compared to the control group. Treatment with Cin inhibited the oxidative stress as it leads to significant reduction in MDA and NO levels of Cin + DFS + OTC, Cin + OTC and Cin + DFS groups relative to DFS + OTC, OTC and DFS exposed groups (*p* < 0.05) (Table 4 and Table 5). Moreover, oral administration of Cin induced a remarkable elevation in the activities of hepatic GSH and SOD, and renal GSH only when compared to the control group (*p* < 0.05). The hepatic and renal activities of GSH, SOD and catalase were significantly reduced in DFS + OTC, OTC, and DFS- treated groups unlike that of the control rats (*p* < 0.05). Whereas, Cin treatment modified the reduction in antioxidant biomarkers caused by DFS + OTC, OTC, and DFS treatments as displayed in (Table 4 and Table 5).

### 3.4. Serum Immunoglobulin, C-Reactive Poteins (CRP) and Cytokines

**Figure 2:**
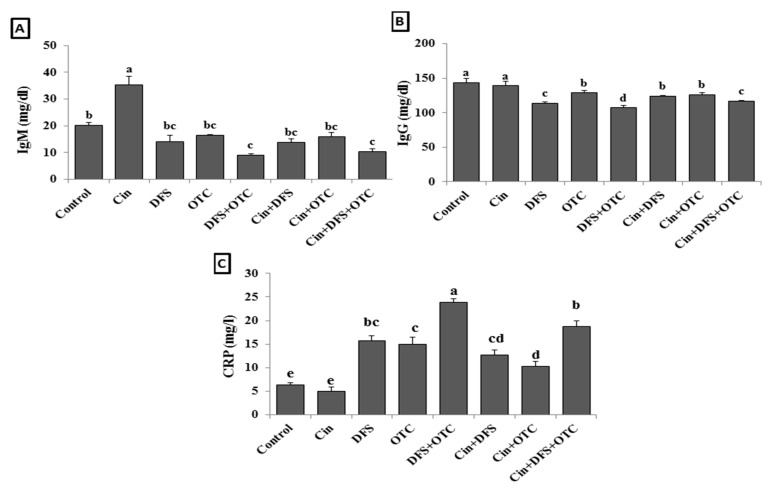
IgM (**A**), IgG (**B**), and CRP (**C**) levels of male albino rats treated orally with cinnamon aqueous extract (Cin, 300 mg/kg b.w./day), single dose of diclofenic sodium (DFS, 100 mg/kg b.w.) and oxytetracycline (OTC, 200 mg/kg b.w./day). Data are expressed as the mean ± SEM (n = 8). Each bar carrying different letters (a, b, c, d, e) is significantly different (*p* < 0.05).

Serum IgM level was significantly elevated in Cin treated group and decreased in DFS + OTC and Cin + DFS + OTC treated groups comparing with the control group (*p* < 0.05). Meanwhile, no significant difference in IgM level was observed in DFS, OCT, Cin + OTC, and Cin + DFS when compared to the control one (*p* < 0.05) (Figure 2A). In addition, the IgG level was significantly reduced and CRP was significantly increased in the DFS + OTC, OTC and DFS groups compared to the control group (*p* < 0.05). The DFS + OTC and DFS groups treated with cinnamon showed improvement in the IgG serum level and reduction in CRP level (but not the return to their normal level) (Figure 2B,C).

**Figure 3:**
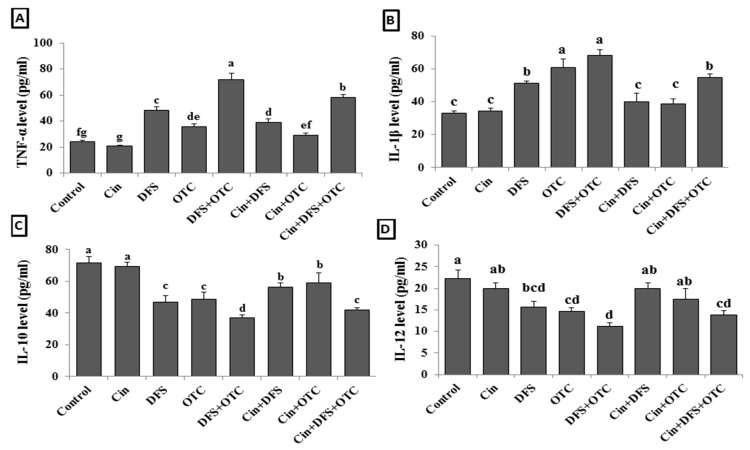
Serum cytokines levels; TNF-α (**A**), IL-6 (**B**), IL-10 (**C**), and IL-12 (**D**) of male albino rats treated orally with cinnamon aqueous extract (Cin, 300 mg/kg b.w./day), single dose of diclofenic sodium (DFS, 100 mg/kg b.w.) and oxytetracycline (OTC, 200 mg/kg b.w./day). Data are expressed as the mean ± SEM (n = 8). Each bar carrying different letters (a, b, c, d, e, f, g) is significantly different (*p* < 0.05).

The elevation of serum TNF-α and IL-1β levels reflect the hepato-renal injury as showed in DFS + OTC, OTC, and DFS groups (*p* < 0.05) (Figure 3B,C). While the treatment of intoxicated rats with Cin showed significantly reduced TNF-α level in DFS + OTC and DFS groups and IL-1β level in DFS + OTC, OTC, and DFS groups (*p* < 0.05) (Figure 2A,B). The serum levels of IL-10 and IL-12 were significantly reduced in DFS + OTC, OTC, and DFS groups when compared with the control one (Figure 3C,D) (*p* < 0.05). The rats that received Cin showed significant elevation in the IL-10 level (*p* < 0.05) (Figure 3C).

### 3.5. Histopathological Assessments

#### 3.5.1. Liver Histopathology

**Figure 4:**
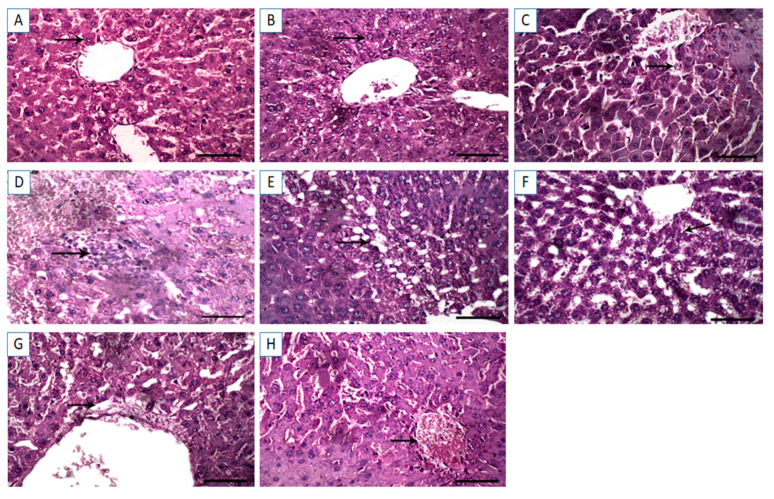
Light photomicrographs of hepatic tissues (sections stained with H&E). (**A**,**B**) control and cinnamon groups: Normal hepatocytes with normal radial arrangement around the central vein. (**C**) DFS group: Necrosis of the hepatocytes with dilatation of the hepatic sinusoids. (**D**) OTC group: Revealing massive hemorrhage replacing hepatic parenchyma and round cells infiltration. (**E**) DFS + OTC group: Vacuolation and necrosis of the hepatocytes. (**F**) DFS + Cin group: Showing remarkable amelioration of histopathological changes compared to DFS. (**G**) OTC + Cin group: Nearly normal hepatocytes with mild perivascular edema, and (**H**) DFS + OTC + Cin group: Hemorrhage replacing hepatic parenchyma. Scale bar = 100 µm.

The hepatic tissue sections from control and Cin treated rats showed normal hepatocytes with normal radial arrangement around the central vein (Figure 4A,B). In contrast, hepatic sections from DFS treated rats displayed necrosis of the hepatocytes with dilatation of the hepatic sinusoids (Figure 4C). Similarly, tissue sections from OTC-treated rats exhibited histopathological alterations, in which massive hemorrhage replacing hepatic parenchyma and round cells infiltration were observed (Figure 4D). Additionally, the histological liver sections of rats treated with both DFS and OTC revealed vacuolation and necrosis of the hepatocytes (Figure 4E). Remarkable amelioration of histopathological changes was noted in the DFS + Cin treated group compared to DFS treated group (Figure 4F). Moreover, histological investigation of group VII (OTC + Cin treated rats) presented nearly normal hepatocytes with mild perivascular edema (Figure 4G). Meanwhile, in the liver of DFS + OTC + Cin treated group, hemorrhage replacing hepatic parenchyma was noted (Figure 4H).

#### 3.5.2. Kidney Histopathology

**Figure 5:**
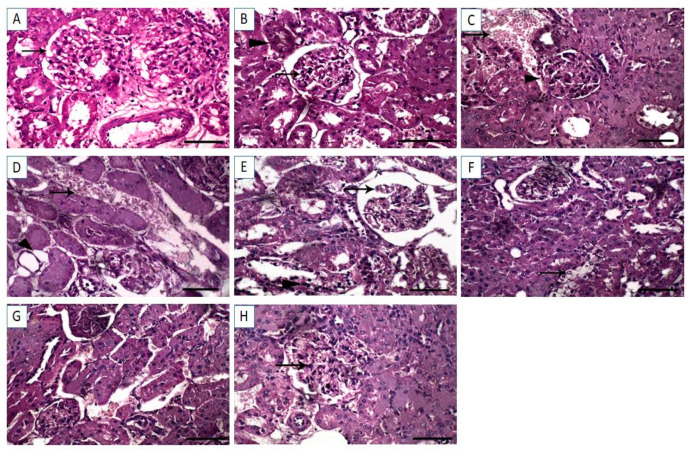
Light photomicrographs of renal tissues (sections stained with H &E). (**A**,**B**) control and cinnamon groups: Normal renal glomeruli and normal renal tubules. (**C**) DFS group: revealed proliferation of the renal glomeruli and marked hemorrhage in interstitial tissue displacing renal parenchyma (**D**) OTC group: Showing hemorrhage in interstitial tissue displacing renal parenchyma. (**E**) DFS + OTC group: Congestion in the renal glomeruli and necrosis in the renal tubular epithelium were observed. (**F**) DFS + Cin group: Degenerative changes in the renal tubular epithelium. (**G**) OTC + Cin group: Fairly normal histological structure, and (**H**) DFS + OTC + Cin group: Revealing mild proliferation of the renal glomeruli and normal renal tubule. Scale bar = 100 µm.

The renal tissue sections from control and Cin groups exhibited normal renal glomeruli and normal renal tubules (Figure 5A,B). However, DFS-treated rats revealed proliferation of the renal glomeruli and marked hemorrhage in interstitial tissue displacing renal parenchyma (Figure 5C). Similarly, the kidney sections from OTC treated rats showed hemorrhage in interstitial tissue displacing renal parenchyma (Figure 5D). Additional renal tissue injury was noted in DFS + OTC treated group, in which congestion in the renal glomeruli and necrosis in the renal tubular epithelium were observed (Figure 5E). In the renal tissue section of DFS + Cin treated group, degenerative changes in the renal tubular epithelium were noted (Figure 5F). Meanwhile, the OTC + Cin treated rats displayed a fairly normal histological structure (Figure 5G). Interestingly, the histological kidney section of DFS + OTC + Cin treated rats showed mild proliferation of the renal glomeruli and normal renal tubules (Figure 5H).

### 3.6. Immunohistochemical Findings

#### 3.6.1. Liver IHC

**Figure 6:**
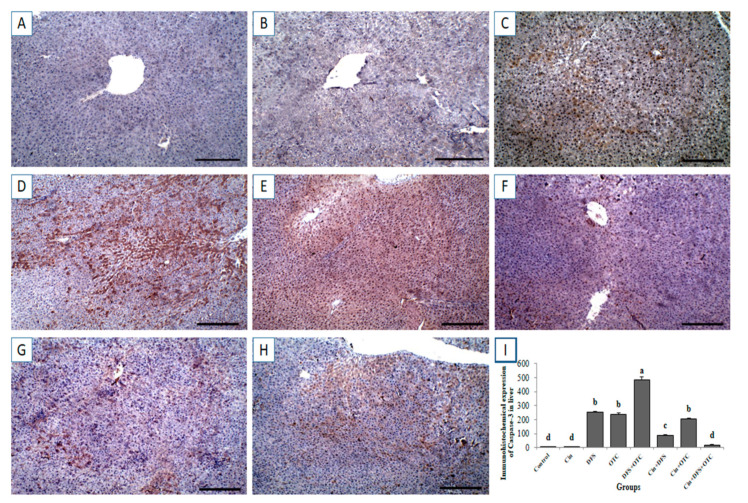
Immunohistochemical staining of rat liver by caspase-3. (**A**,**B**) control and cinnamon groups: Showing negative caspase-3 reaction in the liver sections. (**C**,**D**) DFS and OTC groups: Revealing strong positive caspase-3 reaction in the hepatocytes. (**E**) DFS + OTC group: Showing higher expression levels of caspase-3 relative to those in rats treated with DFS or OTC alone (**F**) DFS+ Cin group: Revealing weak caspase-3 reaction. (**G**) OTC + Cin group: strong caspase-3 positive reaction. (**H**) DFS + OTC + Cin group: Showing reduction in caspase-3 reaction compared to DFS + OTC group. (**I**) Quantification of caspase-3 in the liver tissues in different groups. Data are presented as the mean ± SEM (n = 5), analyzed using one way ANOVA and each bar carrying different letters (a, b, c, d) is significantly different (*p* < 0.05). Scale bar = 100 µm.

**Figure 7:**
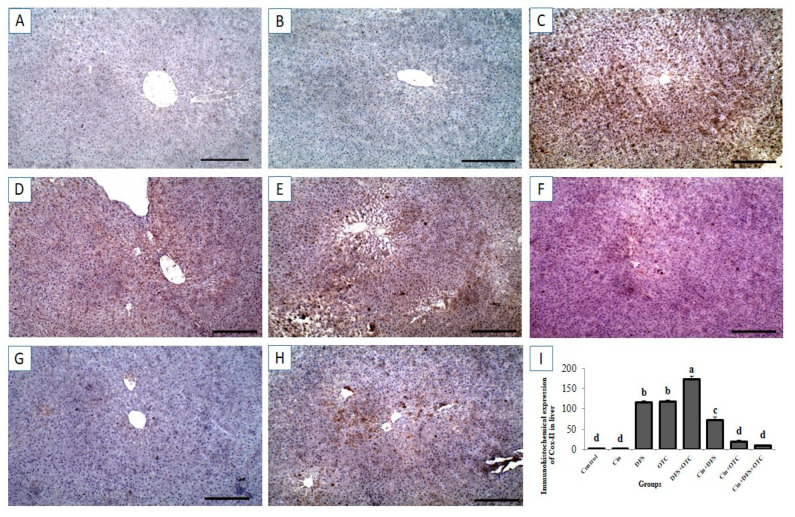
Immunohistochemical staining of rat liver by Cox-II. (**A**,**B**) control and cinnamon groups: Showing negative Cox-II reaction in the liver sections. (**C**,**D**) DFS and OTC groups: Revealing strong positive Cox-II reaction in the hepatocytes. (**E**) DFS+ OTC group: Showing higher expression levels of Cox-II relative to those in rats treated with DFS or OTC alone (**F**) DFS + Cin group: Revealing weak Cox-II reaction. (**G**) OTC + Cin group: Nearly negative Cox-II reaction. (**H**) DFS + OTC + Cin group: Showing reduction in Cox-II reaction compared to DFS + OTC group. (**I**) Quantification of Cox-II in the liver tissues in different groups. Data are presented as the mean ± SEM (n = 5), analyzed using one way ANOVA and each bar carrying different letters (a, b, c, d) is significantly different (*p* < 0.05). Scale bar = 100 µm.

Immunohistochemical investigation revealed negative caspase-3 and Cox-II reactions in the hepatic tissues of control and Cin treated groups (Figure 6A,B, respectively, for caspase-3 and Figure 7A,B, respectively, for Cox-II). On the contrary, strong positive caspase-3 and Cox-II immune staining were noted in the DFS and OTC treated groups (Figure 6C,D, respectively, for caspase-3 and Figure 7C,D, respectively, for Cox-II). Moreover, higher expression levels of capase-3 and Cox-II were observed in rats of the DFS + OTC treated group relative to those in rats treated with DFS or OTC alone (Figure 6E and Figure 7E, respectively). In contrast, weak caspase-3 and Cox-II expression were recorded in DFS + Cin treated rats (Figure 6F and Figure 7F, respectively). Further, OTC + Cin-treated rats displayed strong caspase-3 positive reaction (Figure 6G) and a nearly negative Cox-II reaction (Figure 7G). Interestingly, a reduction in caspase-3 and Cox-II expression was noticed in DFS + OTC + Cin treated group compared to the DFS + OTC treated group (Figure 6H and Figure 7H, respectively).

#### 3.6.2. Kidney IHC

**Figure 8:**
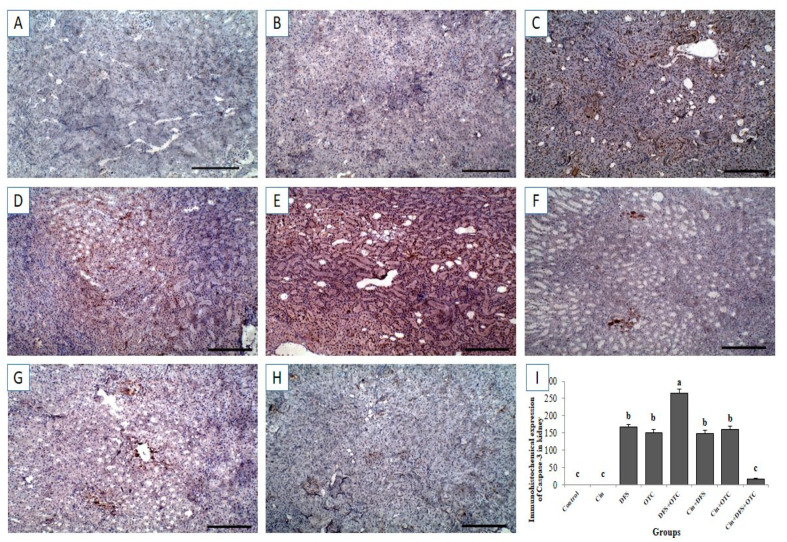
Immunohistochemical staining of rat kidney by Caspase-3. (**A**,**B**) control and cinnamon groups: Showing negative caspase-3 reaction in the renal sections. (**C**,**D**) DFS and OTC groups: Revealing strong positive caspase-3 reaction in the hepatocytes. (**E**) DFS+ OTC group: Showing higher expression level of caspase-3 relative to those in rats treated with DFS or OTC alone (**F**,**G**) DFS + Cin and OTC + Cin group: Revealing reduction in immune reactivity of caspase-3. (**H**) DFS + OTC + Cin group: Showing moderate positive caspase-3 immune staining. (**I**) Quantification of caspase-3 in the kidney tissues in different groups. Data are presented as the mean ± SEM (n = 5), analyzed using one way ANOVA and each bar carrying different letters (a, b, c) is significantly different (*p* < 0.05). Scale bar = 100 µm.

**Figure 9:**
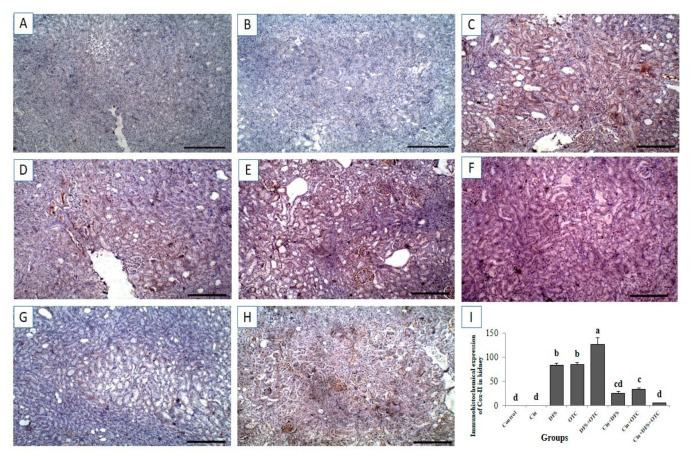
Immunohistochemical staining of rat kidney by Cox-II. (**A**,**B**) control and cinnamon groups: Showing negative Cox-II reaction in the renal sections. (**C**,**D**) DFS and OTC groups: Revealing strong positive Cox-II reaction in the hepatocytes. (**E**) DFS+ OTC group: Showing a higher expression level of Cox-II relative to those in rats treated with DFS or OTC alone (**F**,**G**), DFS + Cin, and OTC + Cin group: Revealing reduction in immune reactivity of Cox-II. (**H**) DFS + OTC + Cin group: Showing moderate positive Cox-II immune staining. (**I**) Quantification of Cox-II in the kidney tissues in different groups. Data are presented as the mean ± SEM (n = 5), analyzed using one way ANOVA and each bar carrying different letters (a, b, c, d) is significantly different (*p* < 0.05). Scale bar = 100 µm.

Immunohistochemistry exhibited negative caspase-3 and Cox-II immunostaining reaction in the renal tissues of control and Cin treated rats (Figure 8A,B, respectively, for caspase-3 and Figure 9A,B, respectively, for Cox-II). On the other hand, strong positive caspase-3 and Cox-II immune staining were recorded in the DFS and OTC treated groups (Figure 8C,D, respectively, for caspase-3 and Figure 9C,D, respectively, for Cox-II). Additionally, DFS + OTC treated group showed higher expression levels of caspase-3 and Cox-II compared to those in the group treated with DFS or OTC individually (Figure 8E and Figure 9E). On the contrary, reduction in immune reactivity of caspase-3 and Cox-II was observed in DFS + Cin treated rats (Figure 7F and Figure 8F, respectively) and in the OTC +Cin treated group (Figure 8G and Figure 9G, respectively). Moreover, DFS + OTC + Cin treated group displayed moderate positive caspase-3 and Cox-II immune staining (Figure 8H and Figure 9H, respectively).

## 4. Discussion

The current study revealed that DFS can exaggerate the hepatorenal toxicity of OTC and that Cin can afford hepatorenal protection against toxicity caused by DFS and OTC, both individually or concurrently. The serum levels of hepatic and renal injury biomarkers provide evidence for the potentiated hepatorenal toxic effect caused by DFS and OTC concurrent therapy. The obtained results declared significantly higher liver enzymes, urea, creatinine, uric acid and lower total protein, albumin and globulin levels in DFS + OTC, OTC, and DFS groups compared to the control group. Similar to our results, El-Maddawy and El-Ashmawy [37], Alabi et al. [7], and Aycan et al. [38] reported that DFS administration was associated with elevated transaminases (AST, ALT, and ALP) that widely used as biomarkers for hepatic injury. Moreover, higher serum urea, and creatinine levels were observed in rats treated with DFS which in turn reflect renal dysfunction as previously reported by Ahmed et al. [10]. The liver injury induced via DFS may be correlated to the metabolic aberration and hypersensitivity which can produce serious liver damage and leaking of cellular enzymes into the blood [39]. Furthermore, Oda et al. [15] demonstrated that intraperitoneal treatment of rats with OTC (200 mg/kg b.w.) for seven days increased serum AST, ALP, total bilirubin, urea, and creatinine decreased serum total protein and albumin that reflect the hepatic and renal damage caused by OTC treatment.

Our histopathological assessment of the liver and kidney sections collected from rats treated with DFS and OTC either individually or concurrently emphasized the obtained findings of hepatorenal dysfunction. Histopathological examination revealed hepatorenal lesions in both the DFS- and OTC treated groups, with the most serious lesions noticed in the combination group. The hepatic histoarchitecture of DFS-treated rats showed necrosis of the hepatocytes with dilatation of the hepatic sinusoids. This is inconsistent with Alabi et al. [7] who observed distortion of the integrity of the liver with marked sinusoidal congestion and perivenular zonal necrosis, in rats exposed to DFS. Further, on histopathological investigation of the kidneys, DFS- treated rats showed proliferation of the renal glomeruli and marked hemorrhage in interstitial tissue displacing renal parenchyma. These findings accord with those of El-Maddawy and El-Ashmawy [37] who reported that treatment of rats with DFS at 13.5 mg/kg once daily for two weeks caused interstitial inflammation and glomerulo-nephritis. Moreover, the histopathological examination of hepatic tissues from the OTC-treated rats exhibited vacuolation and necrosis of the hepatocytes. These findings were parallel to those reported by Letteeron et al. [40] who noted hepatocytic vacuolation, mononuclear cell infiltrates in portal areas, and hepatic necrosis in mice treated with OTC. Additionally, histopathological assessment of the kidneys of OTC-treated group declared hemorrhage in interstitial tissue displacing renal parenchyma. Similarly, Gnanasoundari and Pari [17] announced that treatment of rats with OTC at a dose of 200 mg/kg for 15 days caused alterations in kidney histoarchitecture observed as a focal area of hemorrhage.

Moreover, the immunohistochemical examination of the hepatic and renal tissues demonstrated strong expression of apoptosis marker, caspase-3 in the DFS and OTC treated groups. This result is in line with previous ones which declared that exposure of rats to DFS caused marked increment of caspase-3 protein expression in the liver and kidney enhancing apoptosis of hepatocyte and renal cells [41]. The activation of caspase-3 may be associated with oxidative damage of mitochondria leading to the release of mitochondrial proteins [42,43]. Further, it has been reported that DFS caused over expression of caspase-3 in the liver via affecting lysosomal membrane integrity causing a release of proteolytic enzymes, which increases the mitochondrial membrane permeability and releases of cytochrome c initiating caspase-3.activation and apoptosis [44]. The overexpression of caspase-3 suggested that DFS and OTC induce oxidative stress and inflammation which in turn enhances apoptotic pathways. 

Also, the current investigation demonstrated overexpression of Cox-II, a pro-inflammatory protein; within the hepatocytes and renal cells of DFS and OTC-intoxicated rats. The upregulation of Cox-II in hepatic and renal tissues may be linked to DFS and OTC induced inflammation. Inflammation is regarded as one of the sequelae of oxidative stress [45]. Previous literatures declared that excessive production of free radicals stimulates the synthesis of nuclear factor-kappa B (NF-κB) and other intracellular signaling cascades which in turn stimulate the expression of pro-inflammatory gene as IL-1β, IL-6, TNF-α, and COX-2 [46,47].

Antioxidant defense system has a critical role in the protection of cells from reactive oxygen species (ROS) mediated oxidative injury [5]. Our results showed significantly elevated MDA and NO levels with lower GSH, CAT, and SOD activities of liver and kidney isolated from DFS + OTC, OTC, and DFS treated rats compared to the control rats. Similarly, Boshra and Hussein [48] announced that administration of a single dose of DFS (150 mg/kg body weight) to albino rats caused decrease in hepatic and renal GSH, SOD, and catalase that suggested liver and renal toxicity. The mechanism of DFS-induced oxidative damage in the liver and kidney of treated rats is due to sweeping the antioxidant activities of SOD, CAT, GST, and GSH, enhancing ROS production and deteriorating lipid peroxidation [5]. Further, similar to our study, increased levels of serum and hepatic lipid peroxidation products and decreased antioxidant levels (SOD, catalase, and glutathione peroxidase) were noticed in OTC intoxicated rats (200 mg/kg body weight/day) for 15 days [49]. The oxidative stress of OTC may be owed to hepatic dysfunction, the accumulation of fat droplets in the hepatocytes, lipid peroxidation and generation of ROS [50]. The increased plasma MDA level and reduced blood GSH activity after OTC administration reflected the formation of free radicals and initiation of lipid peroxidation that may be associated with cellular damage [51]. 

Humoral immunity involved the interaction of B cells with the antigen and their subsequent proliferation and differentiation to antibody-secreting plasma cells [52]. There is evidence that IgM and IgG may mediate specific anti-inflammatory signaling pathways [53,54]. The current study showed that IgG level was significantly reduced in the DFS + OTC, OTC and DFS groups while IgM decreased in the DFS + OTC group only compared to the control group. However, limited information is available on the effects of DFS and OTC on serum IgM and IgG levels. This is also confirmed by the lower level of globulins that recorded in the same groups which reflect the hepatic and renal injury caused by DFS and OTC treatment. The increment of gamma-globulin level in the serum of OTC-treated rats may be due to hyperplasia of the reticulo-plasmic tissue of the bone marrow induced by OTC administration [55]. 

The pro-inflammatory cytokines are produced predominantly by activated macrophages and are involved in the up-regulation of inflammatory reactions such as IL-1β, IL-6, and TNF-α, while anti-inflammatory cytokines are a series of immunoregulatory molecules that control the pro-inflammatory cytokine response, such as IL-10 [56]. The higher serum levels of TNF-α and IL-1β and reduction of IL-10 and IL-12 in our results reflect the hepato-renal injury in DFS + OTC, OTC and DFS treated rats. Inflammatory cytokines and chemokines play a main role in nephrotoxicity induced by DFS [57]. These findings are in the same line with that obtained by the previous studies which declared that DFS treatment increased myeloperoxidase (MPO), a critical effector of tissue inflammation, that generate hydrogen peroxides, NO, deleterious hypochlorite levels, and pro-inflammatory cytokines as TNF-α during the inflammation process mediated by DFS [58]. Another study showed that OTC significantly induced the T lymphocyte to secret interferon (IFN-γ) and other cytokines that are involved in the inflammatory responses [59]. 

In the present study, higher total phenolic compounds were found in Cin aqueous extract, followed by flavonoids and tannin contents, respectively. The antioxidant properties of the used extract were also determined in terms of the DPPH assay. Interestingly, the findings of the present study declared that the administration of Cin at 300 mg/kg b.w. ameliorated the hepatorenal injury and oxidative damage, induced by DFS and OTC. In the same vein, Hussain et al. [20] reported that intragastrically pretreatment with 200 mg/kg/day of Cin aqueous extract for 14 days prior to administering a single toxic dose of acetaminophen (200 mg/kg) significantly restores the elevations in ALT and AST levels. This advocates the liver damage repairing and hepatoprotective nature of cinnamon [60]. Other authors confirmed the nephroprotective effects of Cin by reducing serum urea and creatinine levels in rats treated with Cin [26,61]. The ameliorative effect of Cin against liver and kidney oxidative damage may be due to increasing the activity of the antioxidant defense system and scavenging the ROS as well as inhibiting lipid peroxidation [26]. Cinnamon oil was reported to have strong ameliorative effects toward hepatotoxicity, lipid peroxidation (LPO), caspase-3, -9 expression, inflammation (IL-1β, IL-6), DNA fragmentation, and histopathological alterations mediated by acetaminophen [62]. The potential protective activity of Cin may be attributed to its polyphenols and flavonoids contents that act as reactive oxygen and nitrogen species scavengers, redoxactive transition metal chelators, and enzyme modulators [63,64]. Moreover, the active component of Cin as cinnamaldehyde, cinnamic acid, and eugenol are the key of Cin antioxidant and free radicals scavenging properties as their chemical structure consists of hydroxyl groups with or without phenol rings that act as hydrogen atom (H+) donor to neutralize free radicals and stopping the oxidation chain reaction [65,66]. As well, The anti-inflammatory effects of Cin were connected to the presence of various compounds that could suppress the expression of inducible nitric oxide synthesis (iNOS), cyclooxygenase-2 (Cox-2), and nitric oxide (NO) production in different organs [67]. Our histopathological and immunohistochemical findings confirmed the ameliorative effect of Cin against hepatorenal injury caused by DFS and OTC.

Further, our results demonstrated a significantly elevated IgM level in Cin treated group. Also, Cin renovated the reduction in immunoglobulin serum levels induced by DFS + OTC, OTC and DFS treatments. In accordance with our study, Niphade et al. [68] announced that treatment of Swiss albino mice with 10 mg/kg of Cin bark caused an increase in the serum immunoglobulins levels suggesting stimulation of humoral immunity by the active component of Cin (cinnamaldehyde, benzaldehyde, cuminaldehyde, and terpenes). 

Moreover, the current investigation suggested a potent anti-inflammatory function of cinnamon extract that modulates hepato-renal function. The anti-inflammatory effect of Cin may be associated with its various active components including cinnamic aldehyde, cinnamyl aldehyde, tannins. Cin has been shown to have potent anti-inflammatory properties by inhibiting the production of NO, cyclooxygenase (Cox)-II, and prostaglandin (PG) E2 in macrophage cell lines [69]. In addition, dendritic cells cultured with Cin showed significantly decreased expression levels of pro-inflammatory cytokines, such as IL-6, IL-12, IL-17, IFN-γ, and TNF-α, while increasing IL-10 levels [70]. In the same line, Roth-Walter et al. [71] reported that cinnamaldehyde can suppress NF-κB by stabilizing the cell membrane and preventing TLR4 oligomerization as well as down-regulation of the inflammatory and regulatory mediators TNF-α and NO. 

Although the present study discloses for the first time the protective effects of Cin against DFS and/or OTC-induced hepatotoxicity and nephrotoxicity in the rat models, there are some limitations of the present work. Characterization of all Cin active constituents is needed to describe the actions of each derivative exclusively. The evaluation of DNA damage (8-hydroxy-2′-deoxyguanosine) and other apoptotic markers (caspase-7, caspase-9, and poly (ADP-ribose) polymerase (PARP)) following the administration of DFS, OTC, and their combination is considered another limitation in our study, as well as the assessment of other signal transduction pathways involved in downstream cascades, that may be implicated in the ameliorative effects of Cin against DFS and/or OTC toxicity.

## 5. Conclusions

This study revealed that DFS can potentiate the hepatorenal toxic effect of OTC. Therefore, dose regimen adjustment is warranted to avoid this potential adverse effect. Moreover, the study declared that Cin protected the hepatic and renal tissues of rats against toxicity induced by DFS and OTC either individually or concurrently. The hepatorenal protection of Cin may be attributed to its anti-inflammatory properties and to its ability to increase the activity of the antioxidant defense system. Further investigations are necessary to provide additional clinical evidence for the traditional uses of Cin against DNA damage and inflammation mediated by DFS and/or OTC.

## Figures and Tables

**Table 1:** Total phenolic, flavonoid, and tannins contents, and antioxidant activity of the cinnamon aqueous extract.

Phytochemical Constituent	Cinnamon Aqueous Extract
Total Phenols (mg GAE/mL)	10.45 ± 1.12
Flavonoids (mg CE/mL)	4.76 ± 0.43
Tannins (mg GAE/mL)	2.51 ± 0.27
IC_50_ (mg/mL, DPPH)	43.58 ± 3.44

Results are expressed as means ± SEM. GAE; gallic acid equivalents, CE; catechin equivalents.

**Table 2:** Serum levels of hepatic function biomarkers in the control and experimental groups.

Experimental Groups	ALT (U/L)	AST (U/L)	ALP (U/L)	TP (g/dL)	Albumin (g/dL)	Globulin (g/dL)
Control	31.45 ± 1.07 ^e^	48.26 ± 2.18 ^f^	383.08 ± 2.97 ^a^	6.44 ± 0.12 ^a^	3.61 ± 0.20 ^a^	2.84 ± 0.28 ^a^
Cin	33.29 ± 0.50 ^e^	50.59 ± 1.03 ^f^	380.03 ± 2.68 ^a^	6.57 ± 0.16 ^a^	3.54 ± 0.05 ^a^	3.03 ± 0.13 ^a^
DFS	71.95 ± 1.26 ^b^	83.21 ± 2.35 ^c^	483.11 ± 2.43 ^b^	3.99 ± 0.12 ^d^	2.85 ± 0.29 ^b^	1.13 ± 0.39 ^bc^
OTC	66.719 ± 0.98 ^c^	81.70 ± 2.09 ^c^	443.62 ± 5.73 ^c^	5.61 ± 0.23 ^b^	3.12 ± 0.26 ^ab^	2.49 ± 0.27 ^a^
DFS + OTC	85.61 ± 1.96 ^a^	105.53 ± 1.95 ^a^	521.63 ± 2.48 ^a^	3.01 ± 0.17 ^e^	2.67 ± 0.07 ^b^	0.41 ± 0.16 ^c^
Cin + DFS	55.07 ± 1.94 ^d^	74.55 ± 2.39 ^d^	448.65 ± 4.64 ^c^	4.51 ± 0.19 ^c^	2.99 ± 0.15 ^b^	1.52 ± 0.25 ^b^
Cin + OTC	51.78 ± 2.08 ^d^	67.61 ± 0.70 ^e^	418.72 ± 3.06 ^d^	5.69 ± 0.08 ^b^	3.16 ± 0.07 ^ab^	2.53 ± 0.06 ^a^
Cin + DFS + OTC	73.17 ± 1.94 ^b^	97.26 ± 0.67 ^b^	485.43 ± 3.50 ^b^	3.40 ± 0.20 ^e^	2.68 ± 0.08 ^b^	0.73 ± 0.26 ^c^

Data are expressed as the mean ± SEM (n = 8). Means within the same row (in each parameter) carrying different superscripts (a, b, c, d, e, f) is significantly different (*p* < 0.05). Cin, Cinnamon; DFS, Diclofenic sodium; OTC, Oxytetracycline; ALT, alanine aminotransferase; AST, Aspartate aminotransferase; ALP, alkaline phosphatase.

**Table 3:** Serum levels of renal function biomarkers in the control and experimental groups.

Experimental Groups	Urea (mg/dL)	Creatinine (mg/dL)	Uric Acid (mg/dL)
Control	52.25 ± 1.31 ^e^	0.48 ± 0.02 ^e^	2.74 ± 0.20 ^e^
Cin	51.57 ± 1.19 ^e^	0.49 ± 0.04 ^e^	2.66 ± 0.23 ^e^
DFS	80.00 ± 2.35 ^b^	0.76 ± 0.01 ^c^	6.20 ± 0.26 ^b^
OTC	73.51 ± 2.72 ^c^	0.69 ± 0.02 ^d^	4.91 ± 0.43 ^c^
DFS + OTC	90.57 ± 1.71 ^a^	0.98 ± 0.01 ^a^	8.86 ± 0.20 ^a^
Cin + DFS	71.23 ± 1.70 ^c^	0.68 ± 0.02 ^d^	4.38 ± 0.41 ^cd^
Cin + OTC	60.25 ± 4.05 ^d^	0.66 ± 0.01 ^d^	3.91 ± 0.27 ^d^
Cin + DFS + OTC	83.75 ± 0.85 ^b^	0.84 ± 0.02 ^b^	6.24 ± 0.43 ^b^

Data are expressed as the mean ± SEM (n = 8). Means within the same row (in each parameter) carrying different superscripts (a, b, c, d, e) is significantly different (*p* < 0.05). Cin, Cinnamon; DFS, Diclofenic sodium; OTC, Oxytetracycline.

**Table 4:** Hepatic antioxidant and oxidative stress parameters in control and experimental group.

Experimental Groups	MDA (nmol/g. Tissue)	NO (µmol/g. Tissue)	GSH (mg/g. Tissue)	SOD (U/g. Tissue)	Catalase (U/g. Tissue)
Control	53.24 ± 1.11 ^e^	33.53 ± 0.61 ^e^	6.46 ± 0.35 ^b^	435.02 ± 1.47 ^b^	2.42 ± 0.23 ^a^
Cin	42.11 ± 1.42 ^f^	35.71 ± 1.67 ^e^	9.41 ± 0.28 ^a^	470.92 ± 3.34 ^a^	2.32 ± 0.34 ^a^
DFS	87.36 ± 1.71 ^b^	47.47 ± 1.79 ^c^	3.25 ± 0.05 ^d^	402.58 ± 2.51 ^e^	1.14 ± 0.05 ^c^
OTC	71.83 ± 2.85 ^c^	41.98 ± 1.02 ^d^	3.81 ± 0.31 ^d^	410.75 ± 0.80 ^cd^	1.43 ± 0.21 ^bc^
DFS +OTC	97.06±1.01 ^a^	64.96±2.67 ^a^	1.89±0.35 ^f^	374.42±2.20 ^g^	0.59±0.07 ^d^
Cin+DFS	75.71±2.75 ^c^	40.37±1.72 ^d^	4.11±0.05 ^c^	409.17±0.98 ^d^	1.78±0.09 ^b^
Cin+OTC	63.52±1.21 ^d^	36.80 ± 0.89 ^e^	4.82 ± 0.14 ^c^	415.58 ± 0.84 ^c^	1.51 ± 0.19 ^bc^
Cin + DFS + OTC	85.86 ± 0.97 ^b^	55.17 ± 1.29 ^b^	2.56 ±0.24 ^e^	391.25 ± 1.73 ^f^	0.66 ± 0.15 ^d^

Data are expressed as the mean ± SEM (n = 8). Means within the same row (in each parameter) carrying different superscripts (a, b, c, d, e, f) is significantly different (*p* < 0.05). Cin, Cinnamon; DFS, Diclofenic sodium; OTC, Oxytetracycline; MDA, Malondialdehyde; NO, nitric oxide; GSH, reduced glutathione; SOD, superoxide dismutase.

**Table 5:** Renal antioxidant and oxidative stress parameters in control and experimental groups.

Experimental Groups	MDA (nmol/g. Tissue)	NO (µmol/g. Tissue)	GSH (mg/g. Tissue)	SOD (U/g. Tissue)	Catalase (U/g. Tissue)
Control	38.38 ± 1.31 ^e^	37.87 ± 0.17 ^d^	5.22 ± 0.12 ^b^	365.58 ± 3.96 ^a^	1.42± 0.06 ^a^
Cin	30.01 ± 2.80 ^f^	34.39 ± 2.24 ^d^	7.94 ± 0.78 ^a^	372.19 ± 2.08 ^a^	1.62 ± 0.12 ^a^
DFS	68.79 ± 1.89 ^b^	58.96 ± 1.94 ^b^	2.75 ± 0.33 ^de^	333.21 ± 5.44 ^c^	1.15 ± 0.04 ^cd^
OTC	57.91 ± 1.53 ^c^	50.72 ± 2.72 ^c^	4.35 ± 0.13 ^c^	342.15 ± 2.16 ^b^	1.33 ± 0.04 ^bc^
DFS + OTC	78.59 ± 1.49 ^a^	76.21 ± 1.59 ^a^	2.32 ± 0.37 ^e^	326.51 ± 2.47 ^c^	0.67± 0.08 ^e^
Cin + DFS	59.79 ± 2.18 ^c^	51.37 ± 3.09 ^c^	3.72 ± 0.36 ^d^	339.79 ± 0.87 ^b^	1.17 ± 0.09 ^cd^
Cin + OTC	51.17 ± 1.00 ^d^	44.92 ± 1.40 ^c^	4.87 ± 0.45 ^bc^	344.08 ± 2.38 ^b^	1.27 ± 0.06 ^bc^
Cin + DFS + OTC	38.38 ± 1.31 ^e^	63.89 ± 2.36 ^b^	2.69 ± 0.21 ^de^	332.38 ± 1.89 ^c^	0.93 ± 0.09 ^d^

Data are expressed as the mean ± SEM (n = 8). Means within the same row (in each parameter) carrying different superscripts (a, b, c, d, e, f) is significantly different (*p* < 0.05). Cin, Cinnamon; DFS, Diclofenic sodium; OTC, Oxytetracycline; MDA, Malondialdehyde; NO, nitric oxide; GSH, reduced glutathione; SOD, superoxide dismutase.

## Data Availability

The authors confirm that the data supporting the findings of this study are available within the article.

## Abbreviations

Cin	Cinnamon,
DFS	Diclofenic sodium,
OTC	Oxytetracycline,
AST	Aspartate transferase,
ALT	Alanine transferase,
ALP	Alkaline phosphatase,
CAT	Catalase,
GSH	Reduced glutathione,
MDA	Malondialdehyde,
SOD	Superoxide dismutase,
TNF-α	Tumor necrosis factor alpha,
IL-1β	Interleukin-1β,
IL-10	Interleukin-10,
IL-12	Interleukin-12.
